# Supplementary material for: HIPK4 is essential for murine spermiogenesis
Source: eLife. 2020 Mar 12;9:e50209. doi: 10.7554/eLife.50209 (PMC7067585; doi:10.7554/eLife.50209)
Supplement: Supplementary file 1. [file elife-50209-supp1.docx]

| **Key Resources Table** | | | | |
| --- | --- | --- | --- | --- |
| **Reagent type (species) or resource** | **Designation** | **Source or reference** | **Identifiers** | **Additional information** |
| Gene  (*Mus musculus*) | *Hipk4* | Cloned from NIH-3T3 cells | Mm01156517_g1 |  |
| Gene  (*Mus musculus*) | *Hipk4 K40S* | Made in lab |  |  |
| Mouse strain | *Hipk4^+/tm1b^* | Jackson Laboratories | 025579 |  |
| Mouse strain | C57BL/6NJ | Jackson Laboratories | 005304 |  |
| Cell line  (*Homo sapiens*) | HEK-293T | ATCC | CRL-3216 | Cell line used at P3 or P7 |
| Cell line  (*Mus musculus)* | NIH-3T3 | ATCC | CRL-1658 | Cell line used at P3 or P5 |
| Biological sample (*Mus musculus*) | Testis samples | Freshly isolated in lab |  | See Materials and Methods of this paper |
| Biological sample (*Mus musculus)* | Epididymis samples | Freshly isolated in lab |  | See Materials and Methods of this paper |
| Peptide, recombinant protein | PMSG | ProSpec | HOR-272 |  |
| Peptide, recombinant protein | hCG | ProSpec | HOR-250 |  |
| Other | Davidson’s Fixative | Made in lab |  | 30% formaldehyde,15% ethanol, 5% glacial acetic acid, 50% distilled water |
| Antibody | anti-HIPK4  (rabbit polyclonal) | FabGennix International | lot 1651.Pb1.AP | 1:1,000 (WB)  1:50 (IF) |
| Antibody | anti-phosphotyrosine (rabbit polyclonal) | Upstate/Millipore | 4G10 Platinum, 05-1050,  lot 2723728 | 1:1,000 (WB) |
| Antibody | anti-ZP3R/sp56  (mouse monoclonal) | QED Bioscience | 55101  lot 051614-120816 | 1:50 (IF) |
| Antibody | anti-IZUMO1  (rat monoclonal) | Abcam | ab211626  lot GR279965-4 | 1:50 (IF) |
| Antibody | anti-SPACA  (rabbit polyclonal) | Abcam | ab191843  lot GR312512-3 | 1:50 (IF) |
| Antibody | Anti-FLAG M2  (mouse monoclonal) | Sigma-Aldrich | F3165 | 1:50 (IF) |
| Antibody | anti-α-tubulin (rat monoclonal) | Santa Cruz Biotechnology | sc-69970  lot G0109 | 1:50 (IF) |
| Antibody | anti-CAPZA2  (rabbit polyclonal) | ProteinTech | 15948-1-AP | 1:1,000 (WB) |
| Antibody | anti-CAPZA3 (guinea pig polyclonal) | Progen | GP-SH4  lot 804091 | 1:50 (IF) |
| Antibody | anti-CAPZB3 (guinea pig polyclonal) | Progen | GP-SH5  lot 804081 | 1:50 (IF) |
| Antibody | anti-β-actin (rabbit polyclonal) | Cytoskeleton | AAN01  Lot 127 | 1:50 (IF)  1:1,000 (WB) |
| Antibody | anti-DPLY19L2 (guinea pig polyclonal) | Gift from Christophe Arnoult |  | 1:50 (IF) |
| Antibody | anti-SUN1  (guinea pig polyclonal) | Gift from Manfred Alsheimer |  | 1:50 (IF) |
| Antibody | anti-nesperin3 (rabbit polyclonal) | Gift from Arnoud Sonnenberg |  | 1:50 (IF) |
| Antibody | Alexa Fluor 488-conjugated anti-EB3  (rabbit polyclonal) | Abcam | ab203264, lot GR227133-1 | 1:100 (IF) |
| Antibody | HRP-conjugated sheep anti-mouse IgG | GE Healthcare | NA931V, lot 9682503 | 1:20,000 (WB) |
| Antibody | HRP-conjugated donkey anti-rabbit IgG | GE Healthcare | NA934V, lot 9780721 | 1:20,000 (WB) |
| Antibody | Alexa Fluor 594-conjugated anti-guinea pig | Invitrogen | A11076, lot 1924784 | 1:400 (IF) |
| Antibody | Alexa Fluor Plus 647-conjugated goat anti-mouse IgG | Invitrogen | A32728, lot UB275580 | 1:400 (IF) |
| Antibody | Alexa Fluor 647-conjugated goat anti-rat IgG | Invitrogen | A21247, lot 37177A | 1:400 (IF) |
| Antibody | Alexa Fluor Plus 647-conjugated goat anti-rabbit IgG | Invitrogen | A32733, lot TL272452 | 1:400 (IF) |
| Recombinant DNA reagent | pBMN-HIPK4-Y175F-3xFLAG-IRES-mCherry | Made in lab |  | See Materials and Methods of this paper |
| Recombinant DNA reagent | pCL-ECO | Imgenex |  | Retrovirus packing vector |
| Recombinant DNA reagent | pBMN-I-GFP | Gift from Gary Nolan |  | Has IRES-GFP downstream of cloning sites |
| Recombinant DNA reagent | pDONR223 | Invitrogen |  | Gateway Cloning Donor vector |
| Sequence-based reagent | pBMN-3xFLAG-IRES-mCherry-DEST | Made in lab |  | See Materials and Methods of this paper |
| Sequence-based reagent | Genotyping  Murine *Hipk4*  Forward | Integrated DNA Technologies |  | 5’-CCTTTGGCCTTATACATGCAC-3’ |
| Sequence-based reagent | Genotyping  Murine *Hipk4*  Reverse | Integrated DNA Technologies |  | 5’-CAGGTGTCAGGTCTGGCTCT-3’ |
| Sequence-based reagent | Genotyping  Murine *Hipk4 tm1b*  Forward | Integrated DNA Technologies |  | 5’-CGGTCGCTACCATT ACCAGT-3’ |
| Sequence-based reagent | Genotyping  Murine *Hipk4 tm1b*  Reverse | Integrated DNA Technologies |  | 5’-ACCTTGAGATGACCCTCCTG-3’ |
| Sequence-based reagent | Subcloning  Murine *Hipk4*  Forward | Integrated DNA Technologies |  | 5’-CAAAAAAGCAGGCTCAGCCACCATGGCCACCATCCAGTCAGAGACTG-3’ |
| Sequence-based reagent | Subcloning  Murine *Hipk4*  Reverse | Integrated DNA Technologies |  | 5’-CAAGAAA GCTGGGTCGTGGTGCCCTCCAACATGCTGCAG-3’ |
| Sequence-based reagent | Mutagenesis  Murine *Hipk4-K40S* Forward | Integrated DNA Technologies |  | 5’-TCGATCCTGA AGAACGATGCGTACCGAAGC-3’ |
| Sequence-based reagent | Mutagenesis  Murine *Hipk4-K40S* Reverse | Integrated DNA Technologies |  | 5’-GATGGCCACCATTTCACCTGTACTCCGAC-3’ |
| Sequence-based reagent | Gateway cloning Murine *Hipk4*  Forward | Integrated DNA Technologies |  | 5’-GGGGACAAGTTTGTACAAAAAAGCAGGCTCA-3’ |
| Sequence-based reagent | Gateway cloning Murine *Hipk4*  Reverse | Integrated DNA Technologies |  | 5’-GGGGACC ACTTTGTACAAGAAAGCTGGGTC-3’ |
| Sequence-based reagent | Mutagenesis  Murine *Hipk4-Y175F* Forward | Integrated DNA Technologies |  | 5’-CGCTATGTGAAGGAGCCTTTCATCCAGTCCCGCTTCTAC-3’ |
| Sequence-based reagent | Mutagenesis  Murine *Hipk4-Y175F* Reverse | Integrated DNA Technologies |  | 5’-GTAGAA GCGGGACTGGATGAAAGGCTCCTTCACATAGCG-3’ |
| Peptide, recombinant protein | Phusion polymerase | New England Biolabs | M0530L |  |
| Peptide, recombinant protein | PfuUltra II Fusion polymerase | Agilent | 600672 |  |
| Peptide, recombinant protein | BP Clonase II | Invitrogen | 11789020 | Gateway cloning enzyme |
| Peptide, recombinant protein | LR Clonase II | Invitrogen | 11791020 | Gateway cloning enzyme |
| Commercial kit | GeneChip WT Plus Reagent Kit | Thermo Fisher Scientific | 902280 |  |
| Commercial kit | In Situ Cell Death Detection Kit | Sigma-Aldrich | 11684795910 |  |
| Commercial kit | RNAscope 2.5 HD Detection Kit | Advanced Cell Diagnostics | 322370 |  |
| Commercial kit | Trans-Blot Turbo RTA transfer kit, PVDF | Bio-Rad | 1704273 | Midi-size |
| Commercial kit | SuperSignal Femto Kit | Thermo Fisher Scientific | 34095 |  |
| Commercial kit | SuperSignal West Dura Kit | Thermo Fisher Scientific | 34076 |  |
| Commercial kit | Pierce Peptide Quantification Colorimetric Assay | Thermo Fisher Scientific | 23275 |  |
| Commercial kit | Trypsin-LysC protease mix | Promega | V5073 |  |
| Chemical compound, drug | Ca^2+^ ionophore A23187 | Sigma-Aldrich | C9275 | 1,000x stock dissolved in ethanol |
| Software | ImageJ | NIH |  |  |
| Software | Photoshop | Adobe |  |  |
| Software | Transcriptome Analysis Console 4.0 | Thermo Fisher Scientific |  |  |
| Software | SEQUEST | University of Washington |  |  |
| Other | Fluorescein-conjugated peanut agglutinin | Sigma-Aldrich | L7381, lot 046M4030V | 10 µg/mL |
| Other | Alexa Fluor 647-conjugated phalloidin | Invitrogen | A22287, lot 1884190 | 1:400 |
| Other | Vectashield Vibrance mounting medium | Vector Labs | H-1700, ZE1011 |  |
| Other | Prolong Gold Mounting Medium | Thermo Fisher Scientific |  |  |
| Other | Prolong Gold Mounting Medium w/ DAPI | Thermo Fisher Scientific |  |  |
| Peptide, recombinant protein | Collagenase Type I | Worthington Biochemical | LS004194  lot SF8B18091A |  |
| Chemical compound, drug | cOmplete, EDTA-free Protease Inhibitor Cocktail Tablets | Roche | 4693159001 |  |
| Chemical compound, drug | PhosSTOP phosphatase inhibitor | Roche | 4906837001 |  |
| Other | Periodic acid solution | Sigma-Aldrich | 3951 |  |
| Other | Schiff’s reagent | Sigma-Aldrich | 3952016 |  |
| Other | Modified Harris hematoxylin solution | Sigma-Aldrich | HHS32 |  |
| Other | Eosin Y | Sigma-Aldrich | E4009 |  |
