# Supplementary material for: HIPK4 is essential for murine spermiogenesis
Source: eLife. 2020 Mar 12;9:e50209. doi: 10.7554/eLife.50209 (PMC7067585; doi:10.7554/eLife.50209)
Supplement: Transparent reporting form [file elife-50209-transrepform.docx]

***eLife’s* transparent reporting form**

We encourage authors to provide detailed information *within their submission* to facilitate the interpretation and replication of experiments. Authors can upload supporting documentation to indicate the use of appropriate reporting guidelines for health-related research (see [EQUATOR Network](http://www.equator-network.org/%20)), life science research (see the [BioSharing Information Resource](https://biosharing.org/)), or the [ARRIVE guidelines](http://www.plosbiology.org/article/info:doi/10.1371/journal.pbio.1000412) for reporting work involving animal research. Where applicable, authors should refer to any relevant reporting standards documents in this form.

If you have any questions, please consult our Journal Policies and/or contact us: [editorial@elifesciences.org](mailto:editorial@elifesciences.org).

**Sample-size estimation**

- You should state whether an appropriate sample size was computed when the study was being designed
- You should state the statistical method of sample size computation and any required assumptions
- If no explicit power analysis was used, you should describe how you decided what sample (replicate) size (number) to use

Please outline where this information can be found within the submission (e.g., sections or figure legends), or explain why this information doesn’t apply to your submission:

The sample sizes for each experiment are illustrated in the figures themselves, or listed in the figure captions and/or method section. No explicit power analysis was conducted prior to individual studies. Experiments were generally performed three times or using at least three replicates for statistical analysis.

**Replicates**

- You should report how often each experiment was performed
- You should include a definition of biological versus technical replication
- The data obtained should be provided and sufficient information should be provided to indicate the number of independent biological and/or technical replicates
- If you encountered any outliers, you should describe how these were handled
- Criteria for exclusion/inclusion of data should be clearly stated
- High-throughput sequence data should be uploaded before submission, with a private link for reviewers provided (these are available from both GEO and ArrayExpress)

Please outline where this information can be found within the submission (e.g., sections or figure legends), or explain why this information doesn’t apply to your submission:

Information on the number of biological replicates and/or technical replicates is provided in each figure caption and in the Methods section. No data have been excluded.

**Statistical reporting**

- Statistical analysis methods should be described and justified
- Raw data should be presented in figures whenever informative to do so (typically when N per group is less than 10)
- For each experiment, you should identify the statistical tests used, exact values of N, definitions of center, methods of multiple test correction, and dispersion and precision measures (e.g., mean, median, SD, SEM, confidence intervals; and, for the major substantive results, a measure of effect size (e.g., Pearson's r, Cohen's d)
- Report exact p-values wherever possible alongside the summary statistics and 95% confidence intervals. These should be reported for all key questions and not only when the p-value is less than 0.05.

Please outline where this information can be found within the submission (e.g., sections or figure legends), or explain why this information doesn’t apply to your submission:

Most figures show individual data points. If not, the value of N is provided in the figure or figure caption. Errors bars are defined as either SD or SEM in the figure captions, and exact P-values are provided for key experimental comparisons.

(For large datasets, or papers with a very large number of statistical tests, you may upload a single table file with tests, Ns, etc., with reference to sections in the manuscript.)

**Group allocation**

- Indicate how samples were allocated into experimental groups (in the case of clinical studies, please specify allocation to treatment method); if randomization was used, please also state if restricted randomization was applied
- Indicate if masking was used during group allocation, data collection and/or data analysis

Please outline where this information can be found within the submission (e.g., sections or figure legends), or explain why this information doesn’t apply to your submission:

Experimental groups were determined by genotypes (wild-type, heterozygous, or homozygous for the *Hipk4^tm1b^* allele). Animals for different experiments were randomly selected based on age and the timing of the experiment.

**Additional data files (“source data”)**

- We encourage you to upload relevant additional data files, such as numerical data that are represented as a graph in a figure, or as a summary table
- Where provided, these should be in the most useful format, and they can be uploaded as “Source data” files linked to a main figure or table
- Include model definition files including the full list of parameters used
- Include code used for data analysis (e.g., R, MatLab)
- Avoid stating that data files are “available upon request”

Please indicate the figures or tables for which source data files have been provided:

Figure 5. Normalized source data for the microarray analysis is provided as an Excel file entitled Figure 5 – source data 1. The first spreadsheet in the file lists the signal intensities measured for 65,959 genes that were detected in testis samples obtained from wild-type and *Hipk4^-/-^* mice (average of three biological replicates). The second spreadsheet lists genes that exhibited at least a 2-fold difference in expression between genotypes, ranked in order from those that were downregulated in HIPK4-null testes relative to wild-type samples to those that were upregulated.

Figure 6. Mass spectrometry source data for studies of the HIPK4-dependent phosphoproteome in cultured NIH-3T3 cells is provided as an Excel file entitled Figure 6 – source data 1. The first spreadsheet in the file lists all phosphopeptides that were detected in cells expressing either HIPK4, HIPK4-K40S, or HIPK4-Y175F, along with their phosphorylation site, spectral counts, normalized signal intensities, and various calculated scores (A-score, XCorr, PEP-score, etc.). The second spreadsheet lists the phosphopeptides that exhibited at least a 2-fold difference in abundance between cells that overexpress wild-type or mutant HIPK4. The third spreadsheet lists actin-regulators with HIPK4-dependent changes in phosphorylation and indicates their expression levels in germ cells, as previously determined by single-cell RNA-seq (Green, C.D. *et al*. 2018. A comprehensive roadmap of murine spermatogenesis defined by single-cell RNA-seq. *Developmental Cell* **46**: 651-667 e610. DOI: https://doi.org/10.1016/j.devcel.2018.07.025, PMID: 30146481).
